# Trends of the burden of type 2 diabetes mellitus attributable to high body mass index from 1990 to 2019 in China

**DOI:** 10.3389/fendo.2023.1193884

**Published:** 2023-05-31

**Authors:** Jianglin Wang, Lingyun Zhou, Wenjun Yin, Can Hu, Xiaocong Zuo

**Affiliations:** ^1^Department of Pharmacy, The Third Xiangya Hospital, Central South University, Hunan, Changsha, China; ^2^Center of Clinical Pharmacology, The Third Xiangya Hospital, Central South University, Hunan, Changsha, China

**Keywords:** type 2 diabetes, high body mass index, China’s burden of disease, mortality, disability adjusted life year, trends

## Abstract

**Background:**

Overweight and obesity are well-known risk factors for developing type 2 diabetes (T2DM). However, details on the evolution of the T2DM burden attributed to China’s high body mass index (BMI) in China have not been thoroughly studied. This study aimed to investigate the temporal trends of the T2DM burden attributable to a high BMI in China from 1990 to 2019 and to evaluate the independent effects of age, period, and cohort on the burden of T2DM attributed to a high BMI.

**Methods:**

Data on T2DM burden attributable to a high BMI from 1990 to 2019 were obtained from the Global Burden of Disease Study 2019. Deaths, disability-adjusted life years (DALYs), age-standardized mortality rate (ASMR), and age-standardized DALY rate (ASDR) of T2DM attributable to a high BMI were estimated by age and sex. The joinpoint regression model was performed to calculate the annual percentage change (APC) and the average annual percentage change (AAPC) in the burden of T2DM attributed to a high BMI. The age–period–cohort analysis was applied to estimate the independent effects of age, period, and cohort on the temporal trends of mortality and the DALY rate.

**Results:**

In 2019, deaths and DALYs from T2DM attributable to a high BMI in China were 47.53 thousand and 3.74 million, respectively, five times higher than in 1990. Among those under 60 years of age, men had higher deaths and DALYs than women, while the gender differences reversed in those over 60 years of age. Furthermore, the ASMR and ASDR in 2019 were 2.39 per 100,000 (95%UI 1.12–3.90) and 181.54 per 100,000 (95%UI 93.71–286.33), respectively, representing a 91% and 126% increase since 1990. In China, women previously had a higher ASMR and ASDR than men, while the differences in the ASMR and ASDR between the sexes were reversed in recent years. From 1990 to 2019, the ASMR in women increased before 2004 and then decreased from 2004 to 2015, and increased again after, with an overall AAPC value of 1.6%. In contrast, the ASMR in men continued to increase, with an overall AAPC value of 3.2%. The ASDR continued to increase in men and women, with AAPCs of 2.2% and 3.5%, respectively. The age effect showed that the relative risk of mortality increased with age in both men and women, except for the 75–84 age group. The impact of the age on the DALY rate revealed a trend of first rising and then decreasing, peaking at 65–69 years. The effect of the period on the burden of T2DM attributable to a high BMI increased from 1990 to 2019. The cohort effect generally showed a downward trend.

**Conclusion:**

The burden of T2DM attributed to a high BMI in China increased substantially from 1990 to 2019, particularly in men. Therefore, there is an urgent need for gender- and age-based public health guidelines on prevention strategies, early diagnosis, and effective management of T2DM, overweight, and obesity in China.

## Introduction

Diabetes mellitus (DM) is a serious public health problem that significantly influences the lives and well-being of individuals, families, and societies around the world. It is listed as one of the five non-communicable diseases prioritized in the United Nations and WHO Action Plan to address non-communicable diseases (1, 2). The incidence of DM is increasing and has become the eighth leading cause of disease burden in 2019 (3). The International Diabetes Federation Diabetes Atlas study revealed that 536.6 million people aged 20–79 years have diabetes in 2021, with a projected increase to 783.2 million by 2045 (4). Furthermore, DM was responsible for 6.7 million deaths worldwide in 2021, which equates to one diabetes death every 5 s (5). The rising prevalence of DM imposed a significant burden on healthcare systems. In 2021, health expenditures attributable to diabetes were estimated to be 966 billion USD (4).

Among type 1 DM, type 2 DM (T2DM), and gestational DM, T2DM accounts for 90%. According to the most recent study using Global Burden of Disease (GBD) data in 2019, the prevalence, deaths, and disability-adjusted life years (DALYs) of T2DM were 437.9 million, 1,472.9 thousand, and 66.3 million worldwide, respectively (6). With a fifth of the world’s population, China had a rapidly growing prevalence of T2DM and had the highest prevalence, death, and DALY of T2DM in 2019 (6). According to the latest edition of the International Diabetes Federation Diabetes Atlas, there were 140 million adults living with diabetes in China by 2021, accounting for a quarter of the global total, putting tremendous pressure on the public health system (5).

Among numerous metabolic and behavioral risk factors, such as smoking, diet, and physical inactivity, overweight and obesity are the leading contributors to the development and progression of T2DM, as they lead to insulin resistance and beta cell dysfunction (5, 7, 8). Obesity can make it difficult for patients with T2DM to control their weight and blood sugar levels; worsen insulin resistance; aggravate structural and cognitive brain injury; and increase the risk of chronic complications such as diabetic nephropathy, cardiovascular disease, and cerebrovascular disease (9–12). Obesity is also a risk factor for other types of conditions, such as cancers (13). Despite this, the number of obese people continues to rise worldwide. As China’s economy has grown rapidly in the last 30 years, the Chinese lifestyle has changed considerably, with more sedentary behavior and a higher-fat and -energy diet (14). This change has also led to more people being overweight and obese in China.

A recent report on the Nutrition and Chronic Diseases of Chinese Residents (2020) found that the prevalence of diabetes was greater (50%) among adults 18 years or older, a notable increase from 42% in 2015 in the previous 5 years. In addition, according to China’s survey results in 2010, 2013, and 2015–2017, the prevalence of diabetes was 14.3%, 14.7%, and 13.8% among individuals with a BMI range of 25–30 kg/m^2^ and 19.6%, 19.6%, and 20.1% among those with a BMI greater than 30 kg/m^2^, respectively. More striking is that approximately 65% of Chinese people with diabetes are overweight (15, 16). Therefore, evaluating the current landscape and the possible trajectories of the burden of T2DM attributable to a high BMI is crucial for preventing and treating T2DM and overweight in the future.

Although previous studies have reported the latest global spatiotemporal patterns of T2DM burden attributable to high BMI (17), this overall pattern may not properly represent the disease burden in China. Jiang et al. examined the association between the burden of diabetes and high BMI exposure in China, utilizing GBD 2016. Still, this study did not separately address the burden of type 1 DM, T2DM, and gestational DM attributable to a high BMI (18). Comprehensive research has not evaluated long-term trends of T2DM burden attributable to a high BMI in China, specifically concerning the effects of age and sex. Additionally, no study has decomposed the impact of chronological age, period, and birth cohort on the trend of T2DM burden attributable to a high BMI in China. Therefore, this study aimed to investigate the current landscape of the burden of T2DM due to a high BMI and to assess the long-term trend of T2DM burden due to a high BMI in China from 1990 to 2019 using joinpoint regression and age–period–cohort analysis.

## Materials and methods

### Data sources

Data on the burden of T2DM attributable to a high BMI in China were extracted from the GBD 2019 using the Global Health Data Exchange GBD Results Tool (http://ghdx.healthdata.org/gbd-results-tool). The GBD collaborators conducted a comprehensive and systematic evaluation of prevalence and years lived with disabilities for 369 diseases and injuries, mortality for 286 causes, and comparative risks for 87 risk factors by age and sex, covering the period 1 January 1990 and 31 December 2019 in 204 countries and territories, 21 regions, and seven superregions. The general approach to GBD 2019 has been reported in previous studies (3, 19). The epidemiological burden and trend of T2DM attributable to a high BMI in China from 1990 to 2019 was assessed by extracting annual deaths, DALYs, and their corresponding 95% uncertainty intervals (UI)s and age-standardized rates stratified by age (5-year age groups of patients aged 20–94 and ≥95) and sex (both genders, male and female).

### Definitions of type 2 diabetes mellitus and high body mass index

The reference case for T2DM is fasting plasma glucose (FPG) greater than 126 mg/dl (7 mmol/L) or reporting being on drug or insulin treatment for T2DM. Because other measures of blood sugar (glycated hemoglobin A1c, oral glucose tolerance test, and postprandial glucose test), which are inconsistent with the reference case definition, were also accepted to define diabetes in GBD 2019, alternative case definitions used as data inputs were considered and adjusted before beginning the modeling process. The sequelae of T2DM include diabetic neuropathy, foot ulcer, amputation, and retinopathy (moderate and severe vision loss and blindness due to diabetic retinopathy). They are identified by codes E11-E11.1 and E11.3-E11.9 according to version 10 of the International Classification of Diseases (ICD). A high BMI was defined as BMI ≥ 25 kg/m^2^ for adults (age 20+ years).

### Estimation of type 2 diabetes mellitus burden attributed to high body mass index

Previous studies have extensively described fundamental modeling strategies for the GBD 2019 and specific methods to assess the burden of T2DM due to a high BMI (3, 6, 17). Here, we provide a brief overview of approaches to estimate the burden of T2DM due to a high BMI. The first step was data seeking. A comprehensive systematic review of diabetes prevalence, incidence, and mortality was performed in GBD 2019. The epidemiology of diabetes was systematically searched by using the Global Health Data Exchange for multicountry survey programs, national surveys, and longitudinal studies. Meanwhile, additional data that other research groups used to report on the global burden of diabetes, microdata from unpublished national studies, and publications not captured in the PubMed were obtained by enlisting the help of other leaders in the field. Eventually, 1,289 original data sources covering 171 countries were used in the diabetes modeling in GBD 2019. The sequelae of diabetes included diabetic neuropathy and foot ulcers, and amputation due to diabetes was also performed a comprehensive systematic review in GBD 2019. The second step was data inputs, which included estimates of diabetes in a representative population, estimates of mean FPG in a representative population, and individual-level FPG data from surveys and insurance claims in the US and Taiwan. In particular, only 20% of the research provided the type of diabetes estimates, and some studies reporting T2DM separately did not detail the diagnostic criteria. Therefore, the estimates of T2DM were determined by deducting the estimates of type 1 DM from the total estimates of diabetes for age, sex, and location from 1990 to 2019. In addition, the prevalence of obesity per location was used as a covariate. The third step was data processing. To solve the problem of inconsistent sampling and measurement to ensure that data are comparable between different data sources and between high fasting glucose modeling efforts, several data processing steps were performed, including small sample size, mean FPG processing, age splitting, and bias adjustments. The fourth step was modeling. The processed and standardized data were modeled using DisMod MR-2.1, a Bayesian meta-regression tool, to produce the prevalence and age-standardized prevalence rates of diabetes and the sequelae of diabetes for age, sex, geographic location, and year. The proportions extracted from the diabetes sequelae models were multiplied by the parent diabetes model to ensure that all estimates were in the same population space.

The death of a vital registration resource in ICD 10 was used to model T2DM mortality. Data processing for deaths caused by T2DM has also been previously described (3, 6). The Cause of Death Ensemble model was used to estimate T2DM death rates. Years of life lost (YLLs) from T2DM were calculated by multiplying death estimates from T2DM with the corresponding standard life expectancy at the age of death. The prevalence of T2DM sequela multiplied by its disability weight (**Supplementary Table S1**) was used to calculate years lived with disability (YLDs) (3). DALYs for T2DM were calculated as the sum of YLDs and YLLs.

A comprehensive systematic review of the prevalence of a high BMI was also performed. Finally, 2,022 original data sources from 190 countries with a high BMI were entered into the models. The proportion of T2DM burden attributable to a high BMI was estimated using a comparative risk assessment framework, which has been previously described (19). The proportional population attributable fraction (PAF) was used to quantify the contribution of a high BMI to the subsequent occurrence of the T2DM burden.

### Statistical analysis

To assess the magnitude and temporal trend of the burden of T2DM attributable to a high BMI from 1990 to 2019, we employed joinpoint regression analysis, also known as change-point regression, to calculate the annual percentage change (APC), the average annual percentage change (AAPC), and the corresponding 95% confidence intervals (95% CI). As introduced by Kim et al., the joinpoint regression model can be defined as follows (20):


(1) $$\text{log}(y|x)=c+\beta Year+\delta 1{\left(x-\tau 1\right)}^{+}+\cdot \cdot \cdot +\delta_{\text{k}}{\left(x-\tau_{k}\right)}^{+}$$

(2) $$APCi=(e^{b\text{i}}-1)\times 100\%$$

(3) $$AAPC=({\text{e}}^{\frac{\sum b_{i}w_{i}}{\sum w_{i}}}-1)\times 100\%$$

where *y* is the rate indicators, *c* is the constant, *Year* is the observation year, β is the coefficient of year, *τ_k_* is the unknown turning time point (joint point) that needed to be identified, *b_i_* is the estimation of β on the i-th identified trend, and *w_i_* is the length of the i-th identified trend.

If the lower limit of the 95%CI for the related APC/AAPC estimation was higher than zero, there is an increasing trend in the burden of T2DM associated with an increased BMI. Inversely, if the upper limit of the 95% CI for the associated APC/AAPC calculation is less than zero, it suggests a declining trend. If the 95% CI of APC/AAPC includes 0, the trend for the burden of T2DM due to a high BMI is stable.

The age-period-cohort model analysis was used to estimate the impact of age, period, and cohort effects on temporal trends in the mortality and DALY rates of the T2DM attributable to a high BMI. The age-period-cohort model considers age, period, and cohort factors and is commonly used to analyze trends in chronic disease morbidity and mortality and predict future disease burden changes. In the age-period-cohort model, the period effect refers to influence of human factors on the mortality and DALY rates of T2DM due to a high BMI, such as advances in disease diagnosis technology, screening, and early detection; changes in disease definition and registration; and improvements in treatment. All these human factors can affect the mortality and DALY rates of T2DM attributable to high BMI in different periods and produce a period effect. The effect of age is one of the most important determinants of differences in the epidemiology of T2DM, which is the impact of changes in incidence with age. The cohort effect refers to changes in T2DM mortality and DALY rates attributed to a high BMI due to different levels of exposure to risk factors among different generations. Mortality and DALY data for T2DM attributable to a high BMI, population, and period are divided into continuous 5-year intervals from the 20–24 age group to the 90–94 age group and the 95 plus age group, and from 1990–1994 to 2015–2019, respectively. Consecutive 5-year cohorts were defined from 1895–1899 to 1995–1999. The age-period-cohort model is as follows:


$$R=c+\alpha_{\text{i}}A\text{ge}+\beta_{j}P\text{eriod}+\gamma_{k}\text{Cohort}+\text{ϵ}$$

where *R* is the mortality and the DALY rate for the age i age group during the j period; *c* is the intercept term of mortality and the DALY rate; α_i_, β_j_, and γ_k_ are the coefficients of the effects of age, period, and cohort at all levels, respectively; and ϵ is the residual. The exponential values of the coefficients represent the relative risk (RR) of death and DALYs attributed to T2DM for a given age, period, and birth cohort compared to the reference groups.

The Joinpoint Regression Program v4.9.1.0 (April 2022) developed by the US National Cancer Institute Surveillance Research Program was used for the joinpoint regression analysis. Analysis and plot drawing of the age-period-cohort model were performed by R (version 4.2.2) and Stata (version 16.0, StataCorp LP, TX, UA). A two-sided *P-*value less than 0.05 was considered statistically significant.

## Results

### Description analysis of type 2 diabetes mellitus deaths and disability-adjusted life years attributable to a high body mass index in China

**Figure 1:**
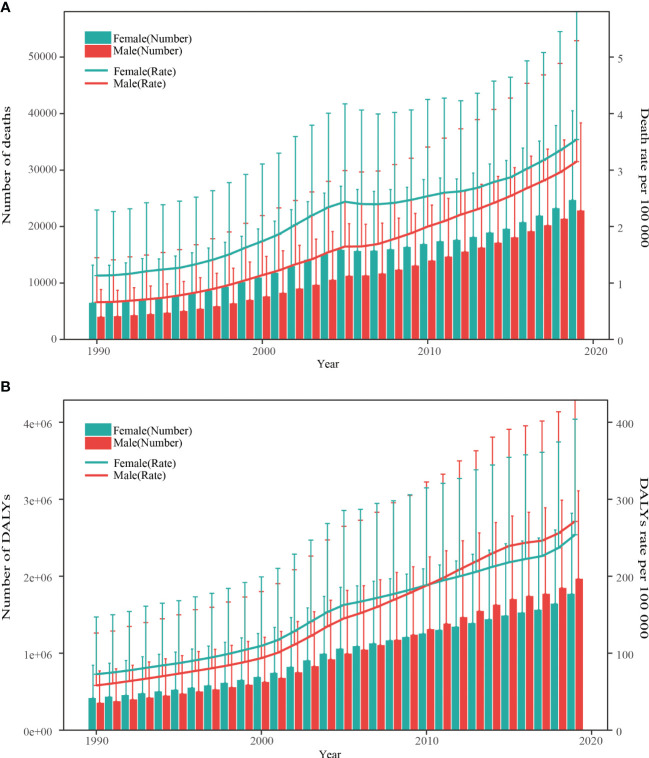
Trends in the all-age number and rate of deaths and DALYs of type 2 diabetes mellitus attribute to high body mass index by sex from 1990 to 2019. **(A)** Death number and rate. **(B)** DALYs number and rate. DALYs, disability-adjusted life years. The vertical lines represent the upper limit of the 95% uncertainty interval.

T2DM deaths and DALYs attributable to a high BMI increased five times in China from 10.51 thousand in 1990 to 47.53 thousand in 2019 and from 0.77 million to 3.74 million in 2019, respectively (**Table 1**). In 2019, the estimated ASMR and ASDR of T2DM associated with a high BMI were 2.39 per 100,000 (95%UI 1.12–3.90) and 181.54 per 100,000 (95%UI 93.71–286.33), respectively (**Table 1**). Between 1990 and 2019, the ASMR and ASDR increased by 91% (95%UI 33%–284%) and 126% (95%UI 60%–365%), respectively (**Table 1**). The ASMR and ASDR of T2DM in 2019 were attributed to a high BMI in proportions of 26.01% (95%UI 12.92%–40.78%) and 38.06% (95%UI 21.09%–55.09%) respectively, as shown in **Table 1**. From 1990 to 2019, the age-standardized proportion of deaths and DALYs from T2DM due to a high BMI had more than doubled (**Table 1**). **Figure 1** shows the trends in deaths and DALYs due to T2DM related to a high BMI in both sexes and all ages from 1990 to 2019. There was a consistent increase in all age group numbers and rates of deaths and DALYs for T2DM related to a high BMI in both men and women over the past 30 years. Furthermore, women consistently had higher death numbers and mortality rates from T2DM attributable to a high BMI than men, but the disparity gradually decreased. The number of DALYs was significantly higher for women than for men before 2008; the opposite was true after that year. The gender gap in all-age DALY rates showed similar trends to the gender gap in the number of DALYs between 1990 and 2019. However, before 2015, the ASMR of T2DM attributable to a high BMI was higher in women than in men, and before 2010, the ASDR of T2DM attributable to a high BMI was also higher in women than in men. After these years, the ASMR and ASDR were lower in women than in men (**Supplementary Figures S1A, B**).

**Table 1:** Deaths and disability-adjusted life years (DALYs) in 1990 and 2019 for type 2 diabetes mellitus (T2DM) attributable to a high body mass index (BMI) in China.

Measure	Year					
	1990			2019		
	Total	Male	Female	Total	Male	Female
Death						
Age-standardized PAF%, (95%UI)	14.45 (4.05, 29.53)	12.30 (2.97, 26.8)	16.01 (4.79, 31.75)	26.01 (12.92, 40.78)	23.65 (11.18, 38.04)	27.69 (14.12, 42.85)
Cases, No.×10^3^ (95% UI)	10.51 (2.94, 21.51)	4.03 (1.00, 8.84)	6.49 (1.91, 13.15)	47.53 (22.51, 76.63)	22.83 (10.50, 38.35)	24.70 (11.55, 40.50)
Mortality per 100,000, No. (95% UI)	0.89 (0.25, 1.82)	0.66 (0.16, 1.45)	1.13 (0.33, 2.29)	3.34 (1.58, 5.39)	3.15 (1.45, 5.29)	3.54 (1.66, 5.81)
Age-standardized rate per 100,000, No. (95% UI)	1.25 (0.34, 2.60)	1.00 (0.24, 2.25)	1.49 (0.43, 3.04)	2.39 (1.12, 3.90)	2.47 (1.10, 4.18)	2.35 (1.09, 3.90)
DALYs						
Age-standardized PAF%, (95%UI)	18.40 (5.25, 36.63)	16.88 (4.39, 34.78)	19.77 (6.07, 38.26)	38.06 (21.09, 55.09)	37.30 (20.49, 54.08)	38.48 (21.28, 55.83)
Cases, No.×10^6^(95% UI)	0.77 (0.21, 1.61)	0.35 (0.09, 0.77)	0.42 (0.12, 0.84)	3.74 (1.91, 5.90)	1.97 (1.00, 3.11)	1.77 (0.93, 2.82)
All-age rate per 100,000, No. (95% UI)	65.20 (17.72, 135.96)	58.12 (14.47, 126.18)	72.74 (21.08, 147.10)	262.77 (134.56, 415.08)	271.31 (138.08, 428.90)	253.90 (133.12, 404.05)
Age-standardized rate per 100,000, No. (95% UI)	80.21 (21.50, 167.40)	71.48 (17.59, 156.52)	88.84 (25.71, 178.47)	181.54 (93.71, 286.33)	195.12 (99.28, 309.13)	167.70 (86.86, 268.54)

DALYs, disability-adjusted life years; PAF, proportional population attributable fraction; UI, uncertainty interval.

**Figure 2:**
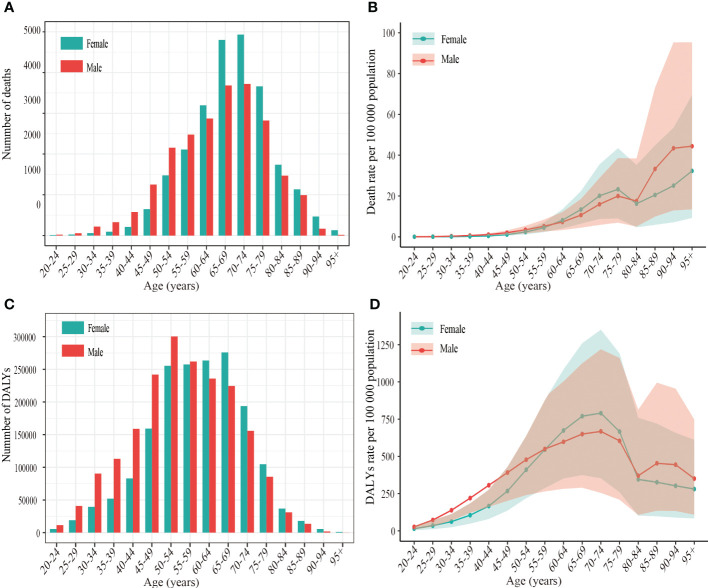
The number and rate of deaths and DALYs of type 2 diabetes mellitus attributable to high body mass index per 100 000 population by age and sex in China, 2019. **(A)** Age-specific number of deaths. **(B)** Age-specific rate of deaths. **(C)** Age-specific number of DALYs **(D)** Age-specific rate of DALYs. DALYs disability-adjusted life years.

The number and rate of deaths and DALYs from T2DM related to high BMI by gender and age group in 2019 are shown in **Figure 2**. In both men and women, the number of T2DM deaths attributable to a high BMI increased with age and peaked in the 70–74 age group; the trend began to decline after this age. The number of T2DM DALYs attributable to a high BMI reached the highest level in the 50–54 age group in men and in the 65–69 age group in women; then, a declining trend was observed in the older age group. The number of deaths and DALYs from T2DM attributable to a high BMI in 2019 for men and women varied substantially by age group. Men had a much higher number of deaths and DALYs than women before the age group of 60–64, while women experienced a significantly higher number of deaths and DALYs than men beyond that age group. Furthermore, except for death rates occurring in adults 75–84 years of age, death rates from T2DM attributable to high a BMI increased with age, starting in the age group of 50–54 for both sexes. Both men and women showed a peak in the T2DM DALY rate attributable to a high BMI in the age group of 70–74 years, followed by a decline in the rate until the age group of 80–84 years for men and the oldest age group for women. There was a substantial difference between men and women in the rate of T2DM mortality and DALY associated with a high BMI.

### Temporal trends for the burden of type 2 diabetes mellitus attributable to high body mass index in China by gender

**Figure 3:**
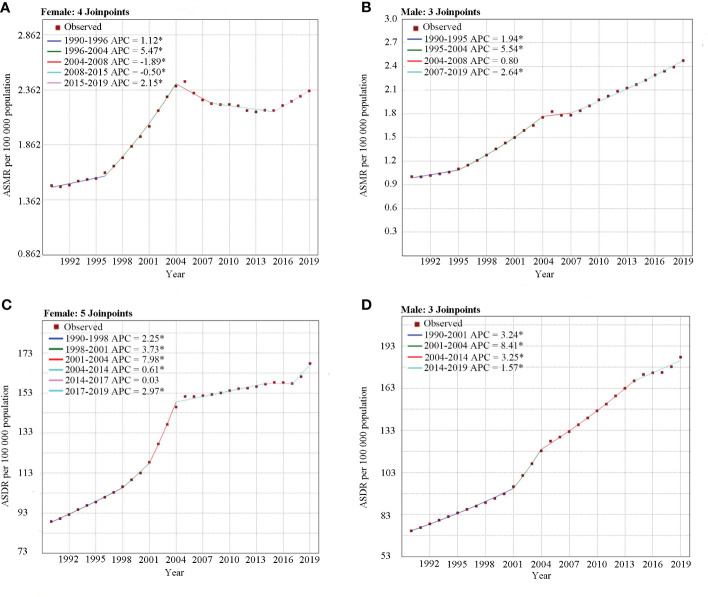
Joinpoint analysis of the ASMR and ASDR for type 2 diabetes mellitus attributable to high body mass index in China from 1990 to 2019 by sex. **(A)** ASMR for females. **(B)** ASMR for males. **(C)** ASDR for females. **(D)** ASDR for males. APC, annual percentage change; ASMR, age-standardized mortality; ASDR, age-standardized disability-adjusted life years rate. *Indicates that the APC is significantly different from zero at the alpha = 0.05 level.

The trends of the ASMR and ASDR for T2DM attributable to a high BMI across all ages from 1990 to 2019 in China are shown in **Figure 3** and **Table 2**. The ASMR and ASDR increased from 1990 to 2019, with AAPC values of 2.3% (95% CI 2.1%–2.5%) and 2.8% (95%CI 2.7%–3.0%), respectively. Interestingly, men had higher AAPCs than women for both the ASMR and ASDR. The ASMR of T2DM attributable to a high BMI in men continuously increased from 1990 to 2019, with three significant increase segments (APC_1990–1995 = _1.9%, APC_2005–2004 = _5.5%, and APC_2007–2019 = _2.6%) and one stage of slight increase (APC_2004–2007 = _0.8).

**Table 2:** The data from joinpoint regression analysis on the age-standardized mortality rate and age-standardized DALY rate of T2DM attributable to a high BMI in both sexes, males and females, from 1990 to 2019 in China.

ASMR			ASDR		
Period	APC,% (95% CI)	AAPC,% (95% CI)	Period	APC,% (95% CI)	AAPC,% (95% CI)
Overall					
1990–1996	1.7 (1.3, 2.0)	2.3 (2.1, 2.5)	1990–2000	2.7 (2.6, 2.7)	2.8 (2.7, 3.0)
1996–2004	5.6 (5.3, 5.8)		2000–2004	7.3 (6.9, 7.8)	
2004–2007	-1.1 (-2.6, 0.4)		2004–2014	2.0 (1.9, 2.1)	
2007–2015	0.9 (0.7, 1.1)		2014–2017	0.6 (-0.1, 1.3)	
2015–2019	2.4 (2.0, 2.9)		2017–2019	2.8 (2.1, 3.4)	
Female					
1990–1996	1.1(0.7, 1.6)	1.6 (1.4, 1.8)	1990–1998	2.2 (2.1, 2.3)	2.2 (2.1, 2.4)
1996–2004	5.5 (5.2, 5.8)		1998–2001	3.7 (2.9, 4.6)	
2004–2008	-1.9 (-2.8, -0.9)		2001–2004	8.0 (7.2, 8.8)	
2008–2015	-0.5 (-0.8, -0.2)		2004–2014	0.6 (0.5, 0.7)	
2015–2019	2.2 (1.6, 2.7)		2014–2017	0.0 (-0.7, 0.6)	
			2017–2019	3.0 (2.3, 3.6)	
Male					
1990–1995	1.9 (1.3, 2.5)	3.2 (3.0, 3.5)	1990–2001	3.2 (3.1, 3.4)	3.5 (3.2, 3.7)
1995–2004	5.5 (5.3, 5.8)		2001–2004	8.4 (5.9, 11.0)	
2004–2007	0.8 (-1.2, 2.9)		2004–2014	3.2 (3.1, 3.4)	
2007–2019	2.6 (2.5, 2.7)		2014–2019	1.6 (1.2, 2.0)	

AAPC, average annual percent change presented for full period; APC, annual percent change; ASMR, age-standardized mortality rate; ASDR, age-standardized disability-adjusted life years rate; CI, confidence interval.

However, the trend for the ASMR in women differs from that of men. Women showed an upward trend during 1990–2004 (APC = 1.1%), 1996–2004 (APC = 5.5%), and 2015–2019 (APC = 2.2%), while a decrease was observed between 2004 and 2008 (APC = −1.9%) and between 2008 and 2015 (APC= −0.5%). Additionally, the joinpoint analysis of the ASDR for T2DM attributable to a high BMI in men exhibited a continuously increasing trend from 1990 to 2019, with the highest increase between 2001 and 2004 (APC = 8.4%). For women, the ASDR trend for T2DM attributable to a high BMI during 1990–2019 indicated that the six segments showed an upward trend, except for a slight decrease from 2014 to 2019 (APC = 0). The highest increase in ASDR in women was observed between 2001 and 2004, with an APC of 8.0%.

### Variation in temporal trends for the burden of type 2 diabetes mellitus attributable to high body mass index in the age, period, and cohort in China by gender

**Figure 4:**
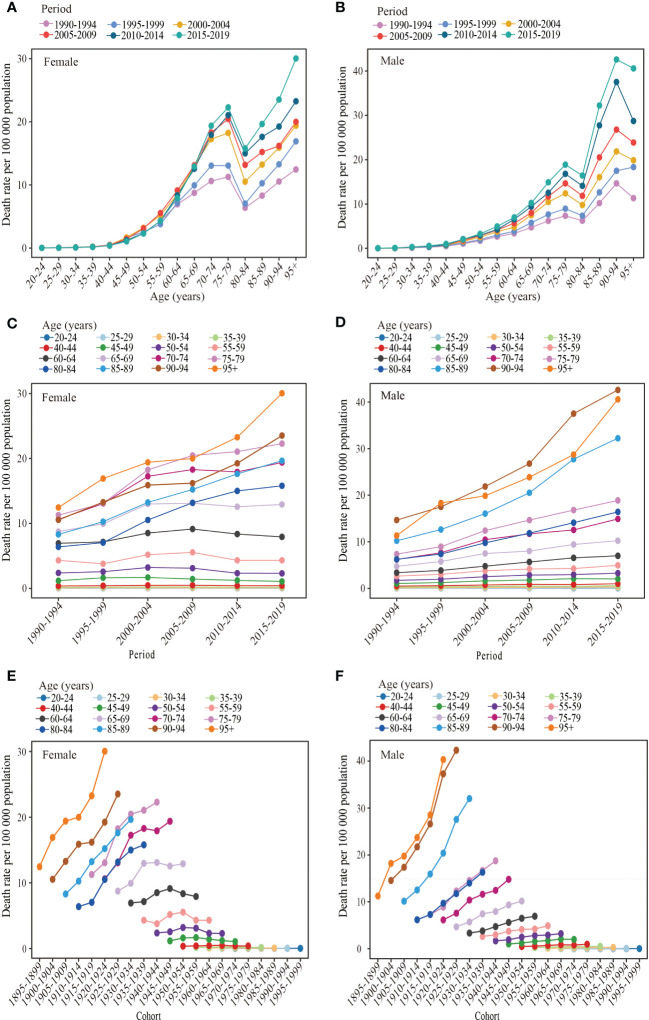
Long-term trends of the mortality rate of type 2 diabetes mellitus attributable to high body mass index in China during 1990-2019 by age, period, and cohort. **(A, B)** Age-specific trends of the mortality rate of type 2 diabetes mellitus attributable to high body mass index for females and males, **(C, D)** Period-based trends of the mortality rate of type 2 diabetes mellitus attributable to high body mass index for females and males. **(E, F)** Cohort-based trends of the mortality rate type 2 diabetes mellitus attributable attributable to high body mass index for females and males.

The age-specific change trend for the mortality rate of T2DM attributable to a high BMI during the observation period is shown in **Figures 4A, B**. The mortality rate of T2DM attributable to a high BMI increased significantly, decreased, and then rose again with age in women from 1990 to 2019. For men, the mortality rate of T2DM attributable to a high BMI showed a decreasing trend with an increase in age among those aged over 90–94 years during 1990–2019, while the mortality rate of other age groups showed a similar trend for women. A rapidly increasing trend was observed between each observation point of mortality from T2DM attributable to a high BMI among those younger than 75–79 years, a decreasing trend was observed for those 75–79 years to 80–84 years, and an increasing trend was observed for those over 80–84 years. The DALY rates of T2DM attributable to a high BMI in both men and women increased and then decreased with age across all periods, with a peak in the 65–74 age group followed by a rapid decline before the age group of 80–84 and then a slower decline (**Supplementary Figures S2A, B**).

The period effects of the mortality rate of T2DM attributable to a high BMI are presented in **Figures 4C, D**. Two types of changes were observed in both women and men in different age groups over time. The first type showed little change in the T2DM mortality rate attributable to a high BMI among people aged 20–64 in women and people aged 20–54 in men during the observation period, indicating a minimal period effect. The second type showed a significant increase in the period effect of the mortality rate of T2DM attributable to a high BMI for women aged over 60–64 years and men aged over 50–54 years between 1990 and 2019. The DALY rates of T2DM attributed to a high BMI in the 20–49 age group for women and in the 20–29 age group for men did not show a significant trend between 1990 and 2019 (**Supplementary Figures S2C, D**). Although the DALY rates of T2DM attributable to a high BMI in other age groups for both women and men showed an overall upward trend, the 70–74 age group had the most significant increase. Furthermore, the rise in DALY rates women with T2DM attributable to a high BMI leveled off after 2005 and showed a downward trend in the 60–69 age group.

The effects of the cohort on the mortality rate of T2DM attributable to high BMI increases are displayed in **Figures 4E, F**. An analysis of birth cohorts revealed that women aged over 60–64 and men aged over 50–54 had a lower mortality rate of T2DM due to a high BMI in the early period compared to the later period. However, for age groups younger than the 60–64 female group and 50–54 male group, the mortality rate of T2DM attributable to a high BMI was less influenced by the birth cohort. **Supplementary Figures S2E, F** indicated that the DALY rates of T2DM attributable to a high BMI in both men and women were lower in the early period than in the later period. However, the trend in DALY rates for T2DM attributable to a high BMI with the passage of the birth cohort varied slightly between women and men. In men, DALY rates increased with birth cohorts, but the effect of birth cohorts on DALY rates was insignificant in the 20–25 age group. For women, DALY rates in the 20–44 and 75–95 age groups increased with birth cohorts, but the increase tended to plateau in the 20–44 age group. However, the DALY rates of T2DM attributable to a high BMI in the 50–74 age group first increased and then decreased with the passage of the birth cohorts.

### The age–period–cohort model results for the burden of type 2 diabetes mellitus attributable to high body mass index in China by sex

**Figure 5:**
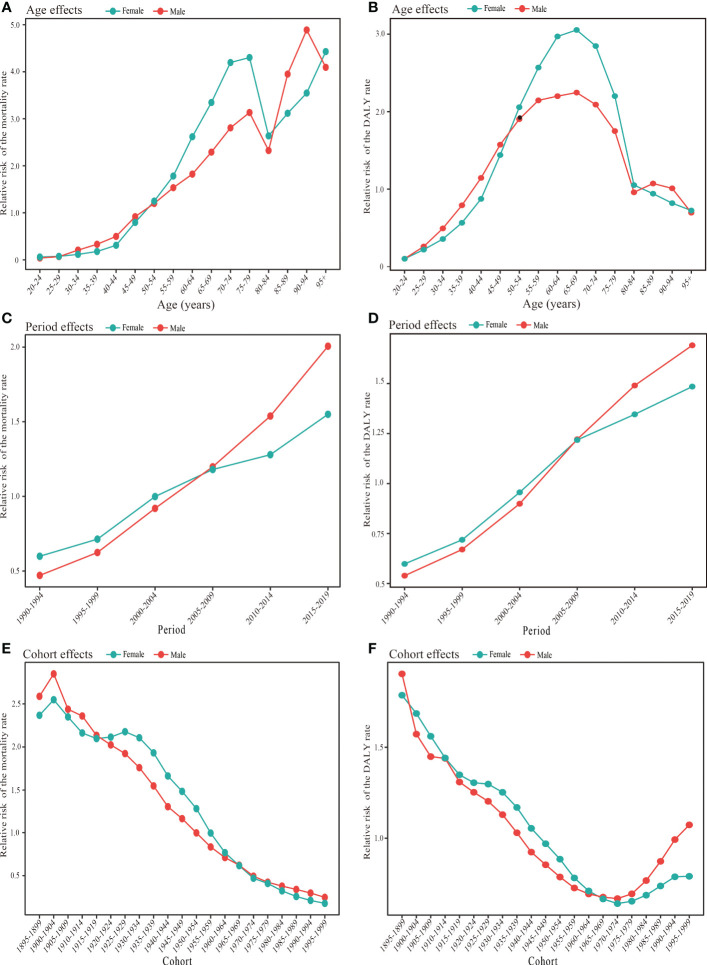
Age, period, and cohort effects on type 2 diabetes mellitus burden attributable to high body mass index in China during 1990-2019 by gender. **(A)** Age effects for types 2 diabetes mellitus mortality rate in females and males. **(B)** Age effects for type 2 diabetes mellitus DALY rate in females and males. **(C)** Period effects for type 2 diabetes mellitus mortality rate in females and males. **(D)** Period effect for type 2 diabetes mellitus DALY rate in females and males. **(E)** Cohort effect for type 2 diabetes mellitus mortality rate in females and males. **(F)** Cohort effects for type 2 diabetes mellitus DALY rate in females and males. DALY, disability-adjusted life year.

**Figure 5** and **Supplementary Table S2** demonstrate that the age, period, and cohort effects impacted the mortality and DALY rates of T2DM attributed to a high BMI for both sexes. After controlling for period and cohort factors, the effect of age on the mortality rates of T2DM attributable to a high BMI showed that the relative risk of mortality generally increased with age for both men and women, except for the 75–84 age group. Calculating the effect coefficients for different age groups revealed that the highest relative risk of death from T2DM attributed to a high BMI occurred in women over 95 years and in men 90–94 years of age, with RRs of 4.43 and 4.89, respectively. Additionally, the age group of 20–24 years had the lowest relative risk of death from T2DM associated with a high BMI, with women experiencing an RR of 0.06 and men experiencing an RR of 0.04. Furthermore, women and men over 50–54 years of age were at risk of T2DM mortality attributable to a high BMI, with an RR > 1. The effect of age on DALY rates showed bell-shaped curves with advancing age in both men and women (**Figure 5B**; **Supplementary Table S2**). An increase with age was observed in the age group under 65–69, followed by a gradual decline. The highest relative risk associated with age for DALY rates was found in the 65–69-year-old group, with an RR of 3.05 (95%CI 3.04–3.07) in women and 2.25 (95%CI 2.22–2.28) in men. The relative risk of T2DM DALY rates attributed to a high BMI was greater than 1 for the age group of 40–79 in men and 45–79 in women.

There was an upward trend in the period effect of T2DM mortality attributed to a high BMI for both women and men between 1990 and 2019 (**Figure 5C**; **Supplementary Table S2**). The age-period-cohort model analysis for the period effect further showed that the RR for men increased more rapidly from 0.47 in the 1990–1994 year group to 2.01 in the 2015–2019 year group than the RR for women from 0.60 in the 1990–1994 year group to 1.55 in the 2015–2019 year group. Women had a relatively higher risk of T2DM mortality attributed to a high BMI than men between 1990 and 2004, while men had a higher risk after the 2000–2004 year group. The 2005–2019 year groups were risk groups with an RR > 1 in mortality in both sexes. Similar trends were observed in the period effect of the T2DM DALY rates attributable to a high BMI (**Figure 5D**; **Supplementary Table S1**).

The cohort effect showed an overall reduction in the risk of T2DM mortality attributed to a high BMI from earlier to later birth cohorts (**Figure 5E**; **Supplementary Table S2**). However, the birth cohort groups of 1895–1954 in women and 1895–1949 in men had a higher relative mortality risk with an RR > 1. In contrast, the effects of the cohort on DALY rates showed a horizontal J-shaped flip, with a decreasing trend in both women and men born before 1974 and an increasing trend in those born after that (**Figure 5F**; **Supplementary Table S2**). In addition, during the birth cohort of 1900–1964, the cohort RR of DALY rates associated with T2DM attributable to a high BMI was slightly lower in men than in women.

### Comparisons of the difference between the burden of type 2 diabetes mellitus attributable to high body mass index in China and in worldwide

**Figure S3** shows the trends in the ASMR and ASDR for T2DM attributable to a high BMI for both sexes combined in China and globally from 1990 to 2019. During this period, the ASMR and ASDR were higher worldwide compared to China, and the difference lasted for 30 years. Furthermore, the ASMR and ASDR for T2DM attributable to a high BMI in China and the world increased from 1990 to 2019.

## Discussion

T2DM is one of the most serious and widespread chronic metabolic diseases that can cause life-threatening complications and disability. It can also result in significant financial costs and reduce life expectancy. In China, the number of people with diabetes has a dramatic increase from 98.4 million in 2013 to 140.9 million in 2020 (21). Obesity is a significant risk factor for T2DM, with over two-thirds of T2DM patients being overweight or obese. The prevalence of overweight and obesity in adults aged 18 years or older increased substantially from 16.4% and 3.6% in 1992 to 34.3% and 16.4% in 2015–2019 in China, respectively (22, 23). This study evaluated the temporal trends of the T2DM burden attributable to a high BMI in China from 1990 to 2019 using joinpoint regression and age-period-cohort analysis. We found that the absolute numbers of death and DALYs in T2DM attributable to a high BMI in China were 47.53 thousand and 3.74 million in 2019, respectively, approximately five times higher than that in 1990. However, the total population only increased by 24.34% from 1990 to 2019 in China, indicating a faster growth rate of the burden of T2DM attributable to a high BMI than that of the country’s population growth, which accurately represented a continuously increasing burden of T2DM attributable to a high BMI. Furthermore, a roughly one-fold increase in age-standardized mortality and DALY rates of T2DM attributed to a high BMI. The trends in the ASMR and ASDR of T2DM attributed to a high BMI in China from 1990 to 2019 were similar to the worldwide. The significant increase in the burden of T2DM due to a high BMI can be attributed to multiple factors, such as changes in dietary patterns, aging and population growth, urbanization, and environmental pollution (24).

The burden of T2DM caused by a high BMI increased significantly for both men and women between 1990 and 2019 but grew more for men. In addition, the burden of T2DM attributable to a high BMI used to be consistently higher for women than men, but the difference has decreased or even changed in recent years. The factors that led to this phenomenon are not fully understood. The patterns of changes in the prevalence rates of overweight and obesity among men and women were similar to the changes in the burden of T2DM attributed to a high BMI between sexes. In China, men previously had a lower prevalence of overweight and obesity than women, but this gap has shrunk or even been reversed in recent years. According to the 2002 China National Nutrition Surveys (CNNSs), the prevalence of overweight and obesity among adults was 30.3% in women and 29.6% in men. However, in the 2010–2012 CNNSs, the prevalence values were 41.6% in women and 42.4% in men, consistent with the trend of increasing disparity between women and men in the prevalence of obesity observed in the China Chronic Disease and Risk Factor Surveillance (25–27). Other possible explanations could be gender differences in treatment and control rates for T2DM. Although the rate of T2DM treatment in Chinese men doubled in 2017 compared to the previous year, the control rate did not increase significantly. Both rates have improved in women since 2010 (28). Furthermore, bariatric surgery has been rising in China in recent years. However, the proportion of obese patients undergoing bariatric surgery is highly uneven in China, with women accounting for 75.1% and men only 24.9% (29). Such sex differences in bariatric surgery may explain the sex disparity in the burden of T2DM attributable to a high BMI. These findings highlight the need for targeted policies and interventions that address the growing trend and gender disparities in the burden of T2DM attributed to a high BMI in China. Such measures should be implemented properly and appropriately based on gender-specific considerations.

The number of deaths and DALYs of T2DM attributable to a high BMI was primarily concentrated in age groups 50–89 and 40–79, respectively, consistent with other studies (17). Our analysis also shows that the number of deaths and DALYs of T2DM due to a high BMI was higher in men than in women in age groups<60 years, while it is lower in men than in women in age groups ≥60 years, consistent with global data (17). Unhealthy lifestyle behaviors, such as smoking and alcohol consumption, are more prevalent among men than women in China, which is an essential factor contributing to the higher burden of T2DM due to a high BMI among men than among women in age groups<60 years. The age-dependent sex difference in the burden of T2DM due to a high BMI could be attributed to the combined effect of age-dependent sex differences in T2DM and obesity. According to The China Diabetes Atlas, there was no significant difference in the prevalence of T2DM between different genders before 2002, with rates high and low in both men and women (28). However, since the 21st century, the prevalence of T2DM in Chinese men has been higher than in women. Another study has shown that the sex disparity in the incidence of T2DM fluctuates throughout life, and women experience a higher prevalence than men during youth. In contrast, men have higher rates of T2DM than women in midlife, and the incidence of T2DM is roughly equivalent between the sexes in later life (30). Insulin clamp studies also show that men have stronger resistance to insulin than women in late puberty and as adults (31). In addition, women who develop T2DM typically have a higher BMI than men. The average age-adjusted BMI of women at the time of diagnosis is 1.8 kg/m^2^ higher than men’s (32, 33). As a result, women can have insulin resistance and metabolic dysfunction for a long time before they are diagnosed with T2DM, putting women with diabetes at increased cardiovascular risk than men with diabetes (34). Combined with the fact that women have a longer life expectancy than men, this could explain why the burden of T2DM attributable to a high BMI is higher in older women than in older men (35).

This study used joinpoint regression analysis to evaluate changes in the ASMR and ASDR of T2DM attributable to a high BMI in China. From 2004 to 2016, the trend for the ASMR and ASDR for women decreased slightly and increased slightly, respectively. However, both the ASMR and ASDR in men showed a substantial increase from 2004 to 2016, which may have been driven by rapid social, economic, and environmental transitions and cultural factors. First, China has made significant efforts to prevent and control obesity since 2003, issuing various policies, recommendations, and guidelines (36–38). Meanwhile, China has made significant efforts and advances in diabetes prevention, risk factor management, self-management education and support, and the integration of medical care modalities. However, there is always an opportunity for improvement, especially in rural areas with limited healthcare services and education access. Second, the perception of the female body image in Chinese society has favored a lean body type in recent years.

In contrast, a larger body size for men is often viewed as a symbol of strength and masculinity, influenced by long-standing social and cultural norms. In addition, as China continues to modernize, physical activity and labor intensity decrease in urban and rural areas and significantly more for women than for men (39). However, unlike the trend in most countries, the prevalence of physical inactivity in China in recent years has been significantly higher for men at 16% than for women at 12% (40). One possible reason for the lower rate of physical inactivity among women compared to men in China is the popularity of “square dancing,” a type of fitness activity that has gained widespread popularity among middle-aged and elderly women in recent years. Square dancing is an excellent way to get women to exercise, especially in rural areas, where finding other exercise methods can be challenging. Additionally, square dancing has been shown to have social and mental health benefits, which can further contribute to its popularity among women (41, 42).

Using the age-period-cohort method, we further examined the effects of age, period, and cohort factors on the mortality and DALY rates of T2DM attributable to a high BMI in China. Age effects are typically defined as modifications caused by changes in physical, psychological, and social status resulting from biological age changes. As expected, the effect of age on T2DM mortality due to a high BMI generally increases with age. The 10th edition of the International Diabetes Federation Diabetes Atlas showed that age was a significant and independent risk factor for T2DM, with a similar trend expected to continue until 2045 (5). In addition, the prognosis is also adversely affected by the fact that T2DM complications such as cardiovascular disease, neuropathy, retinopathy, and nephropathy are more prevalent and worse in older patients. However, the effect of age on the DALY rate exhibited bell-shaped curves as the age increased. This may be related to the lower prevalence of overweight and obesity among older adults in China. Previous epidemiological evidence has shown that obesity and overweight generally increased with age, but later in adulthood, there was a slight decrease (43).

The period effect refers to the risk of morbidity or mortality caused by changes in natural conditions or social environments during a specific period. This is reflected mainly in the differences in disease risk caused by the changes in medical levels, diagnosis and treatment technology, health knowledge, and economic and cultural factors. Similarly, the effect of the period on mortality and DALY rates of T2DM attributable to a high BMI showed an increasing trend throughout the period, indicating that the period effect played a significant role in the growing trend of the burden of T2DM attributable to a high BMI. Over 40 years of reform and opening, China’s economy, science and technology, overall quality of life, and medical services have all improved significantly. With the improvement of the living standard of Chinese residents, the dietary structure has changed. However, these have also brought new health threats to the Chinese people, mainly including environmental pollution and destruction, changes in lifestyle, the intrusion of large amounts of synthetic chemicals into human life, and mental health problems.

China is changing from a high-carb, high-fiber, and low-fat diet to a high-fat, high-energy, and low-carb diet (44). The daily dietary energy supply per capita in China has also increased significantly, from 2,100 to 2,400 kcal in the early 1980s to 3,000–3,100 kcal in the early 2010s, which is the direct cause of the increase in the overweight and obese population (44). In addition, unhealthy processed foods are rising and becoming more accessible and affordable (45). These trends have increased non-communicable diseases such as diabetes, hypertension, and cardiovascular disease. In the last 40 years, with the improvement in the consumption level and the lifestyle transformation, the sports time of Chinese residents decreased rapidly and the sedentary time gradually increased. With the pace of urbanization, modernization, and informatization, people are spending more and more times sitting in front of their desks. The proliferation of private cars, computers, smartphones, and other devices that make people’s lives more convenient has dramatically reduced the amount of daily physical activity. Cable TV, internet entertainment, fast video streaming, mobile games, e-sports, and other forms of entertainment also increase the time people spend sitting. Furthermore, due to the improvement of medical treatment and the popularization of diabetes screening in China in recent years, an increasing number of diabetes cases are being screened at an early stage, which is one of the reasons for the growing burden of T2DM attributable to a high BMI.

The cohort effect represents the impacts specific to one’s age group, those born in the same calendar year due to exposure to the same cultural, environmental, and social changes. Our findings showed that the RR of the cohort effect on T2DM mortality and DALY rates attributable to a high BMI have decreased, suggesting that earlier birth cohorts have a higher burden of T2DM attributable to a high BMI than later birth cohorts. This phenomenon may be related to better education and increased health awareness in the later cohorts than in the earlier cohorts. Furthermore, a previous study indicated that the group with unfortunate experiences in early life would have poorer physical health in adulthood (46). Studies have shown that pregnant women who experience nutritional deprivation during pregnancy are particularly vulnerable and that the physical health of their children is often shaped throughout their lives by their mothers’ experiences of starvation during pregnancy. As adults, these children have relatively high levels of triglycerides and low-density lipoprotein cholesterol and are at greater risk of developing diseases such as obesity and diabetes (47).

According to these findings, multiple policy changes and interventions are needed to reverse the increasing trend of the burden of T2DM attributable to a high BMI in China. Fortunately, some initiatives are already in place, such as the National Basic Public Health Service Program and the Healthy China 2030 Plan, which aims to improve the prevention and management of T2DM and obesity. However, more efforts are needed to ensure its effective implementation and sustainability. Therefore, it is crucial to engage all stakeholders, including governments, civil society organizations, and the private sector, in the implementation process and to establish monitoring and evaluation mechanisms to track progress and identify areas for improvement. First, we should improve public awareness and education on chronic diseases such as diabetes and obesity, increase public awareness of healthy lifestyles, and encourage people to improve their ability to care for themselves. Second, the food safety administration department should strengthen the supervision of food production, processing, sales, and other links and establish a food safety system. Third, the education and health departments must jointly strengthen nutrition education; promote a balanced diet; reduce the intake of foods high in sugar, fat, and salt, and increase the intake of vegetables and fruits. Fourth, the healthcare sector should establish a health management mechanism, regular physical examinations, and health counseling for high-risk groups. At the same time, a chronic disease management system should be installed. Collaboration between doctors, nurses, nutritionists, and other healthcare professionals allows for comprehensive treatment and management of patients. Fifth, researchers should strengthen basic and applied research on diseases such as T2DM and obesity. This will enable a solid a scientific foundation and technical support to prevent and treat these conditions.

This study has the following limitations: (1) it was a secondary analysis of data collected from the GBD 2019 study. Therefore, the general limitations of GBD studies, such as potential bias, are unavoidable, which can lead to some degree of deviation from the actual situation. However, the 2019 GBD study has applied robust statistical methods to solve this problem; (2) the GBD data are updated slowly, currently only up to 2019. Therefore, the use of previous data may not reflect the latest disease trends; 3) the burden of T2DM attributable to a high BMI could not be divided into the burden of T2DM due to overweight and obesity due to the lack of relevant data; (4) complications from T2DM are not taken into account in estimating the burden of T2DM attributable to a high BMI; (5) the study does not include children and individuals aged<20 years; 6) we did not take in account the difference in the burden of T2DM attributable to a high BMI between different provinces, different socioeconomic development, different ethnic groups, and rural and urban groups in China since these data were not available. Future research should focus on a comparative analysis of these factors to guide the development of appropriate health policies and programs and promote the realization of Healthy China 2030; and 7) the age-period-cohort model only considers the population level and ignores individual differences, which could lead to an ecological fallacy when extrapolating the overall trend to individuals.

## Conclusions

T2DM attributable to a high BMI has caused a serious disease burden for the Chinese population, mainly among middle-aged and older adults. The burden of T2DM attributable to a high BMI has increased significantly in China in the last 30 years and has grown more in men than in women. According to the joinpoint analysis, the men’s ASMR and ASDR of T2DM due to a high BMI increased approximately linearly from 1990 to 2019, while the women’s ASMR and ASDR fluctuated. Between 2004 and 2015, there was even a decrease in the ASMR among women. The relative risk of T2DM mortality attributable to a high BMI continued to increase with age and period but decreased with the birth cohort. However, the effects of age and cohort on the DALY rate showed a bell-like and a flip horizontal J-shaped curve trend, respectively, but the impacts of the period on the DALY rate gradually increased. Our findings will provide a better epidemiological basis for future T2DM and high BMI management. The growing burden of T2DM due to a high BMI in China urgently requires close collaboration between policymakers, researchers, and healthcare professionals to develop gender- and age-based public health guidelines on prevention strategies, early diagnosis, and effective management of T2DM, overweight, and obesity. Additionally, education campaigns on healthy lifestyle choices and regular physical activity must be promoted to prevent the onset of T2DM, overweight, and obesity.

## Data availability statement

The original contributions presented in the study are included in the article/**Supplementary Material**. Further inquiries can be directed to the corresponding author.

## Ethics statement

Ethical review and approval was not required for the study on human participants in accordance with the local legislation and institutional requirements. Written informed consent for participation was not required for this study in accordance with the national legislation and the institutional requirements.

## Author contributions

J-LW and X-CZ designed the study and interpreted the results. J-LW analyzed the data and performed the statistical analysis. X-CZ supervised the study. W-JY and L-YZ double-checked all the data. J-LW, CH and X-CZ wrote the initial manuscript. All authors contributed to the article and approved the submitted version.
