# Supplementary figures and images for: Trends of the burden of type 2 diabetes mellitus attributable to high body mass index from 1990 to 2019 in China
Source: Front Endocrinol (Lausanne). 2023 May 31;14:1193884. doi: 10.3389/fendo.2023.1193884 (PMC10264794; doi:10.3389/fendo.2023.1193884)

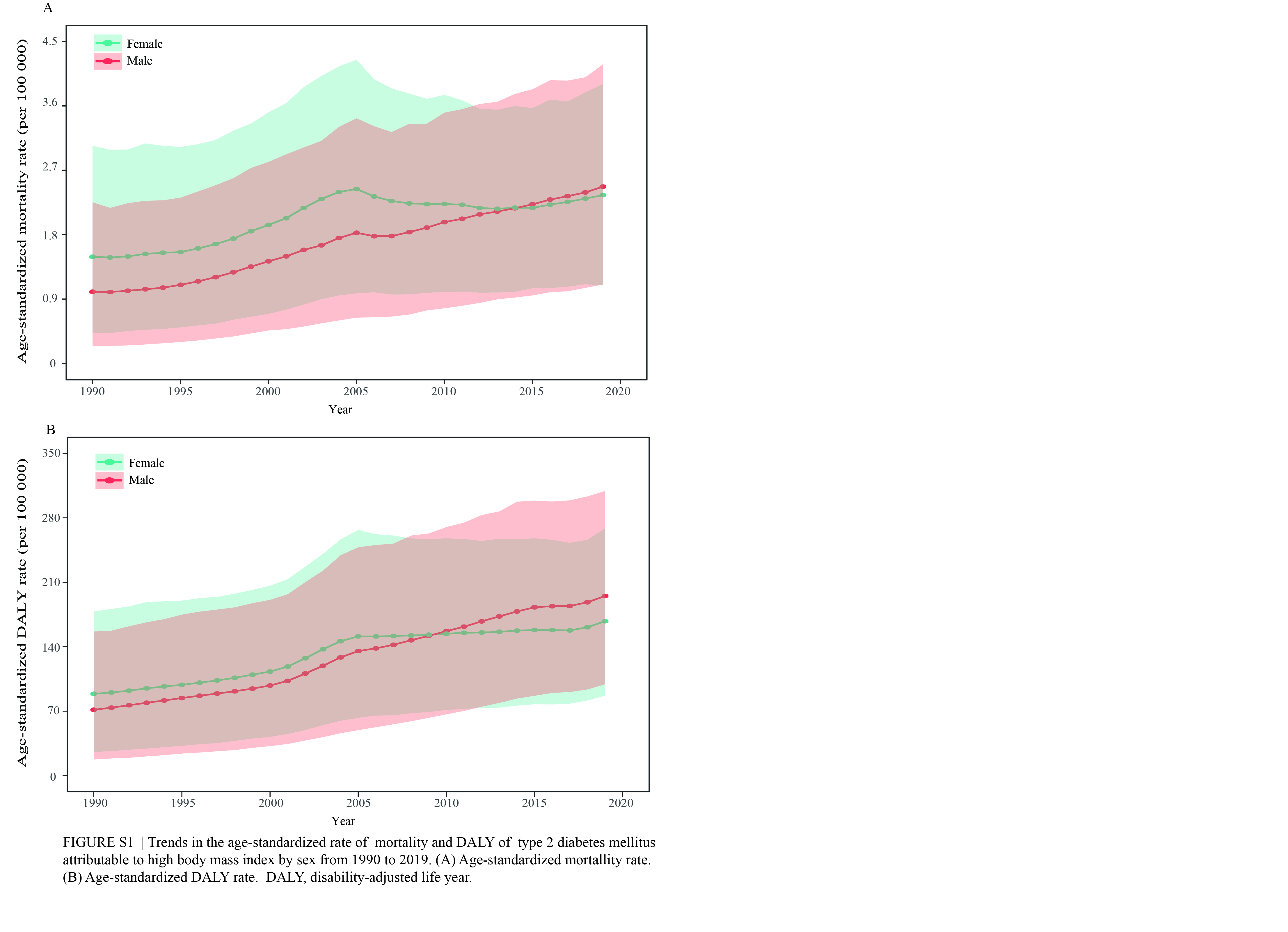

Supplement: Supplementary file 1 [file Image_1.jpeg]

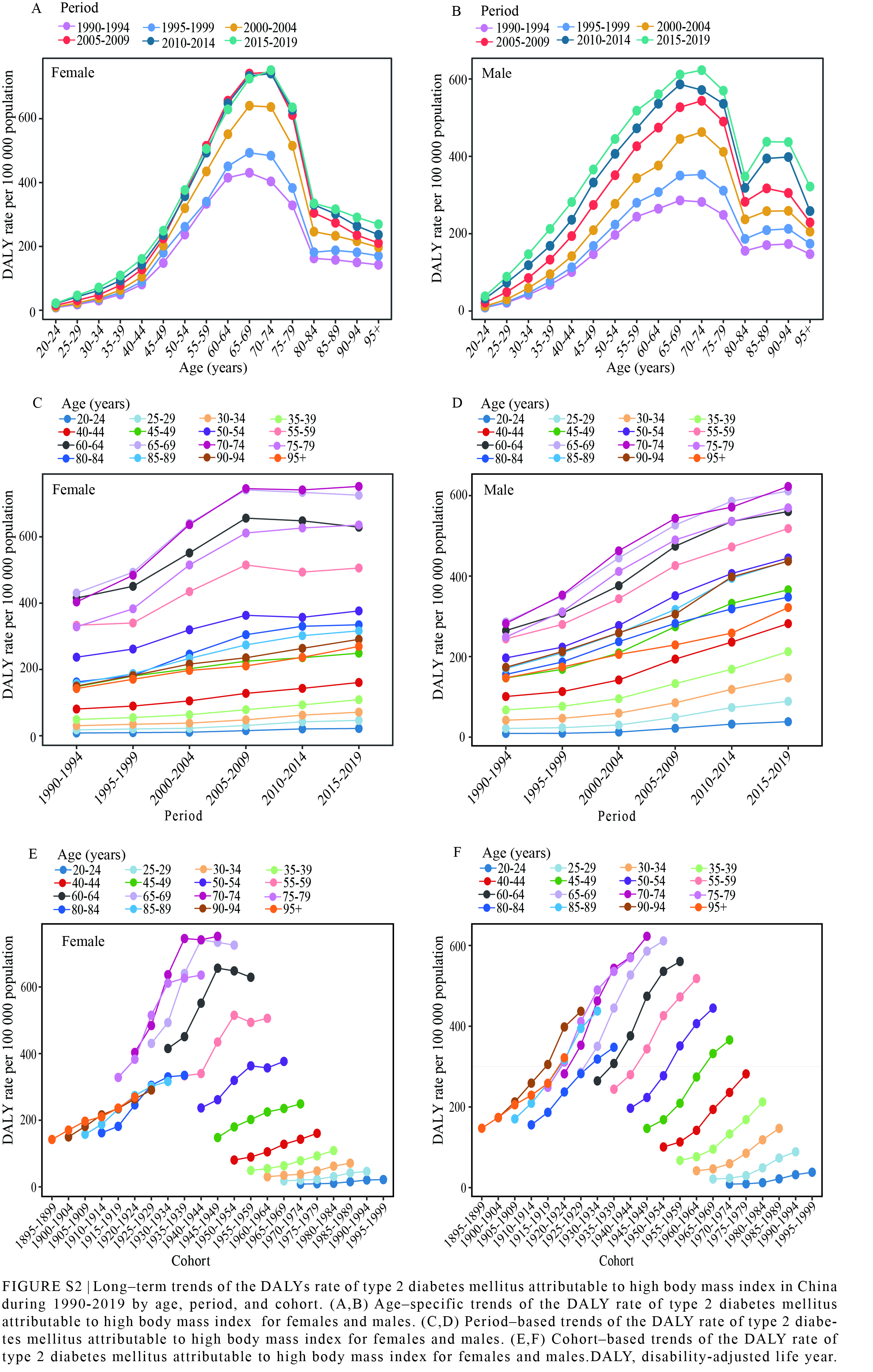

Supplement: Supplementary file 2 [file Image_2.jpeg]

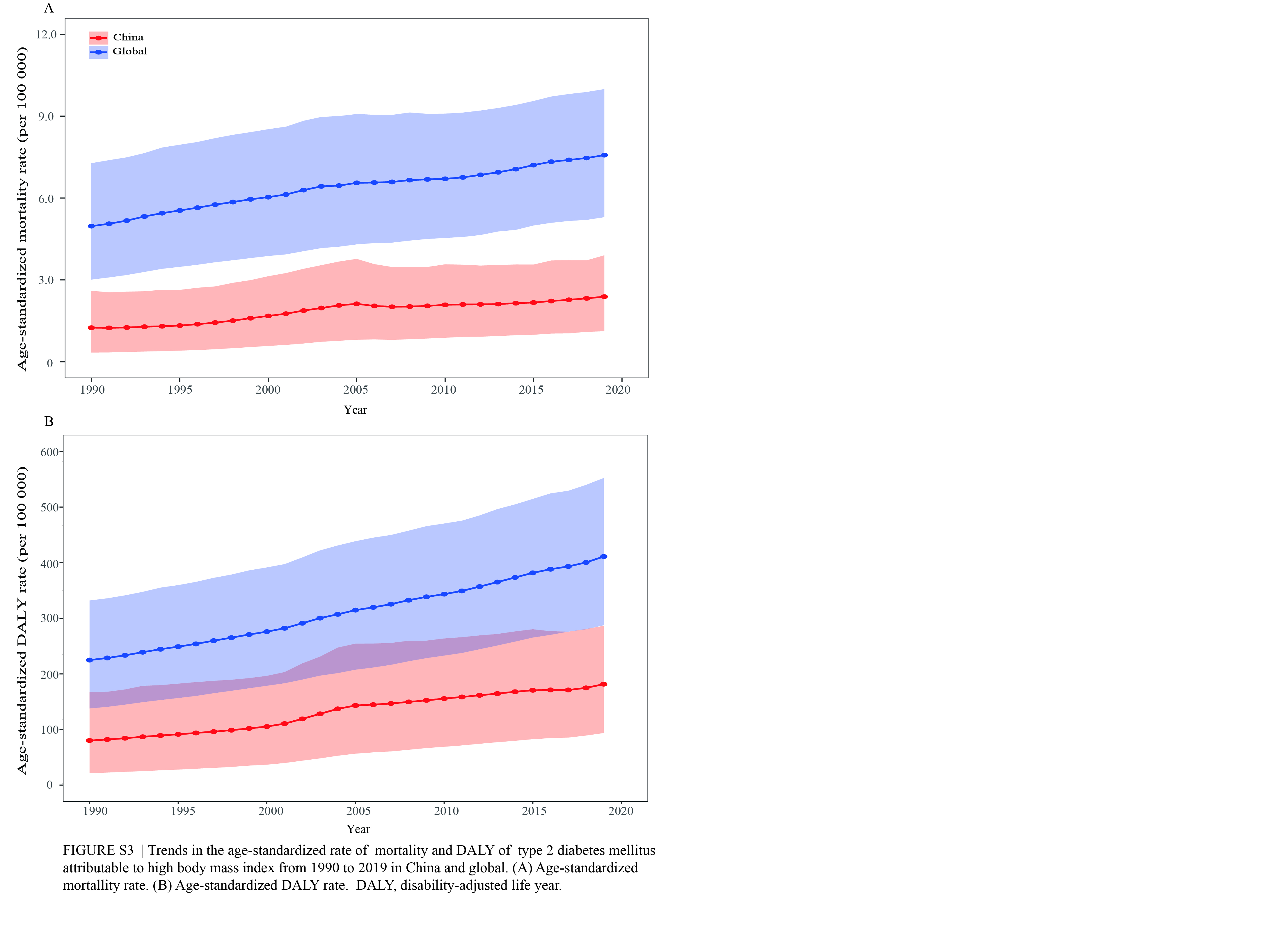

Supplement: Supplementary file 3 [file Image_3.jpeg]
