# Supplementary material for: Trends of the burden of type 2 diabetes mellitus attributable to high body mass index from 1990 to 2019 in China
Source: Front Endocrinol (Lausanne). 2023 May 31;14:1193884. doi: 10.3389/fendo.2023.1193884 (PMC10264794; doi:10.3389/fendo.2023.1193884)
Supplement: Supplementary file 4 [file Table_1.docx]

**TABLE S1** The severity levels and associated disability weights applied to type 2 diabetes-related outcomes

| Severity level | Disability weights (95% CI) |
| --- | --- |
| Uncomplicated diabetes mellitus | 0.049 (0.031- 0.072) |
| Diabetic neuropathy | 0.133 (0.089- 0.187) |
| Diabetic neuropathy with diabetic foot | * |
| Diabetic neuropathy with treated amputation | * |
| Diabetic neuropathy with untreated amputation | * |
| Moderate vision loss due to diabetes mellitus | 0.031 (0.019-0.049) |
| Severe vision loss due to diabetes mellitus | 0.184 (0.125-0.259) |
| Blindness due to diabetes mellitus | 0.187 (0.124-0.26) |

*Disability weights were generated by a combination of two health states: neuropathy and diabetic foot/amputation. CI, confidence interval.
