# Supplementary material for: Trends of the burden of type 2 diabetes mellitus attributable to high body mass index from 1990 to 2019 in China
Source: Front Endocrinol (Lausanne). 2023 May 31;14:1193884. doi: 10.3389/fendo.2023.1193884 (PMC10264794; doi:10.3389/fendo.2023.1193884)
Supplement: Supplementary file 5 [file Table_2.docx]

**TABLE S2** Age-period-cohort (APC) model analysis results for mortality and DALY rates of type 2 diabetes mellitus attributable to high body mass index in China by gender

| Variable case | Female | | | | Males | | | |
| --- | --- | --- | --- | --- | --- | --- | --- | --- |
|  | Mortality rate | | DALY rate | | Mortality rate | | DALY rate | |
|  | Coefficient | Relative risk | Coefficient | Relative risk | Coefficient | Relative risk | Coefficient | Relative risk |
| Age | | | | | | | | |
| 20-24 | -2.82(-3.01, -2.63) | 0.06(0.05, 0.07) | -2.29(-2.31, -2.27) | 0.10(0.10, 0.10) | -3.27(-3.51, -3.02) | 0.04(0.03, 0.05) | -2.28(-2.32, -2.24) | 0.10(0.10, 0.11) |
| 25-29 | -2.61(-2.75, -2.46) | 0.07(0.06, 0.09) | -1.52(-1.53, -1.50) | 0.22(0.22, 0.22) | -2.69(-2.87, -2.51) | 0.07(0.06, 0.08) | -1.35(-1.38, -1.31) | 0.26(0.25, 0.27) |
| 30-34 | -2.16(-2.27, -2.04) | 0.12(0.10, 0.13) | -1.03(-1.05, -1.02) | 0.36(0.35, 0.36) | -1.58(-1.71, -1.45) | 0.21(0.18, 0.24) | -0.71(-0.74, -0.68) | 0.49(0.48, 0.51) |
| 35-39 | -1.73(-1.82, -1.64) | 0.18(0.16, 0.19) | -0.57(-0.58, -0.56) | 0.57(0.56, 0.57) | -1.11(-1.22, -1.00) | 0.33(0.30, 0.37) | -0.23(-0.26, -0.21) | 0.79(0.77, 0.81) |
| 40-44 | -1.17(-1.25, -1.10) | 0.31(0.29, 0.33) | -0.13(-0.14, -0.13) | 0.87(0.87, 0.88) | -0.7(-0.78, -0.61) | 0.50(0.46, 0.54) | 0.14(0.12, 0.16) | 1.15(1.12, 1.17) |
| 45-49 | -0.23(-0.28, -0.18) | 0.79(0.75, 0.84) | 0.36(0.36, 0.37) | 1.44(1.43, 1.45) | -0.09(-0.15, -0.02) | 0.92(0.86, 0.98) | 0.45(0.44, 0.47) | 1.57(1.55, 1.60) |
| 50-54 | 0.22(0.18, 0.27) | 1.25(1.19, 1.31) | 0.72(0.72, 0.73) | 2.06(2.05, 2.07) | 0.18(0.13, 0.23) | 1.2(1.14, 1.26) | 0.64(0.63, 0.65) | 1.90(1.88, 1.92) |
| 55-59 | 0.58(0.54, 0.62) | 1.78(1.72, 1.85) | 0.94(0.94, 0.95) | 2.57(2.56, 2.58) | 0.43(0.39, 0.47) | 1.53(1.47, 1.60) | 0.76(0.76, 0.77) | 2.15(2.13, 2.16) |
| 60-64 | 0.96(0.93, 0.99) | 2.62(2.53, 2.70) | 1.09(1.08, 1.09) | 2.97(2.96, 2.98) | 0.6(0.56, 0.64) | 1.82(1.75, 1.90) | 0.79(0.78, 0.80) | 2.20(2.18, 2.22) |
| 65-69 | 1.21(1.18, 1.24) | 3.35(3.24, 3.45) | 1.12(1.11, 1.12) | 3.05(3.04, 3.07) | 0.83(0.78, 0.88) | 2.29(2.18, 2.41) | 0.81(0.80, 0.82) | 2.25(2.22, 2.28) |
| 70-74 | 1.43(1.40, 1.47) | 4.2(4.06, 4.34) | 1.05(1.04, 1.05) | 2.85(2.82, 2.87) | 1.03(0.97, 1.10) | 2.81(2.63, 3.00) | 0.74(0.72, 0.76) | 2.09(2.05, 2.13) |
| 75-79 | 1.46(1.42, 1.50) | 4.3(4.13, 4.48) | 0.79(0.78, 0.80) | 2.2(2.18, 2.22) | 1.14(1.06, 1.23) | 3.13(2.88, 3.41) | 0.56(0.54, 0.58) | 1.75(1.71, 1.79) |
| 80-84 | 0.97(0.92, 1.02) | 2.64(2.51, 2.77) | 0.05(0.04, 0.06) | 1.05(1.04, 1.07) | 0.84(0.74, 0.95) | 2.32(2.09, 2.58) | -0.04(-0.07, -0.01) | 0.96(0.93, 0.99) |
| 85-89 | 1.14(1.07, 1.20) | 3.12(2.93, 3.32) | -0.06(-0.08, -0.04) | 0.94(0.93, 0.96) | 1.37(1.25, 1.50) | 3.95(3.48, 4.48) | 0.07(0.03, 0.11) | 1.07(1.04, 1.11) |
| 90-94 | 1.27(1.18, 1.35) | 3.55(3.27, 3.85) | -0.20(-0.22, -0.18) | 0.82(0.80, 0.84) | 1.59(1.42, 1.75) | 4.89(4.14, 5.78) | 0.01(-0.04, 0.06) | 1.01(0.96, 1.06) |
| 95+ | 1.49(1.36, 1.62) | 4.43(3.88, 5.06) | -0.32(-0.36, -0.28) | 0.72(0.70, 0.75) | 1.41(1.08, 1.74) | 4.09(2.95, 5.69) | -0.36(-0.47, -0.26) | 0.70(0.63, 0.77) |
| Period | | | | | | | | |
| 1990-1994 | -0.51(-0.54, -0.48) | 0.6(0.58, 0.62) | -0.51(-0.52, -0.51) | 0.60(0.59, 0.60) | -0.75(-0.81, -0.69) | 0.47(0.44, 0.50) | -0.62(-0.63, -0.60) | 0.54(0.53, 0.55) |
| 1995-1999 | -0.34(-0.36, -0.31) | 0.71(0.70, 0.73) | -0.33(-0.33, -0.33) | 0.72(0.72, 0.72) | -0.47(-0.51, -0.43) | 0.62(0.60, 0.65) | -0.40(-0.41, -0.39) | 0.67(0.66, 0.68) |
| 2000-2004 | 0(-0.02, 0.02) | 1(0.98, 1.02) | -0.05(-0.05, -0.04) | 0.96(0.95, 0.96) | -0.08(-0.11, -0.06) | 0.92(0.90, 0.94) | -0.11(-0.11, -0.10) | 0.90(0.90, 0.90) |
| 2005-2009 | 0.17(0.15, 0.18) | 1.18(1.16, 1.20) | 0.20(0.19, 0.20) | 1.22(1.22, 1.22) | 0.18(0.16, 0.20) | 1.20(1.17, 1.22) | 0.20(0.20, 0.20) | 1.22(1.22, 1.23) |
| 2010-2014 | 0.25(0.23, 0.27) | 1.28(1.25, 1.31) | 0.30(0.29, 0.30) | 1.35(1.34, 1.35) | 0.43(0.40, 0.47) | 1.54(1.49, 1.59) | 0.40(0.39, 0.41) | 1.49(1.48, 1.50) |
| 2015-2019 | 0.44(0.41, 0.47) | 1.55(1.51, 1.59) | 0.40(0.39, 0.40) | 1.48(1.48, 1.49) | 0.70(0.64, 0.75) | 2.01(1.90, 2.12) | 0.53(0.51, 0.54) | 1.69(1.67, 1.72) |
| Cohort | | | | | | | | |
| 1895-1899 | 0.86(0.22, 1.51) | 2.37(1.24, 4.52) | 0.58(0.39, 0.77) | 1.79(1.48, 2.16) | 0.95(-0.75, 2.65) | 2.59(0.47, 14.18) | 0.64(0.17, 1.12) | 1.90(1.18, 3.06) |
| 1900-1904 | 0.94(0.66, 1.21) | 2.55(1.94, 3.35) | 0.52(0.45, 0.60) | 1.69(1.56, 1.82) | 1.05(0.51, 1.59) | 2.85(1.67, 4.88) | 0.45(0.30, 0.61) | 1.57(1.35, 1.84) |
| 1905-1909 | 0.85(0.69, 1.02) | 2.35(2.00, 2.77) | 0.45(0.40, 0.49) | 1.56(1.50, 1.63) | 0.89(0.57, 1.21) | 2.44(1.77, 3.36) | 0.37(0.28, 0.46) | 1.45(1.33, 1.58) |
| 1910-1914 | 0.77(0.65, 0.9) | 2.16(1.91, 2.45) | 0.37(0.33, 0.40) | 1.44(1.40, 1.49) | 0.86(0.60, 1.12) | 2.36(1.81, 3.07) | 0.36(0.29, 0.44) | 1.44(1.34, 1.55) |
| 1915-1919 | 0.74(0.64, 0.84) | 2.1(1.90, 2.32) | 0.30(0.27, 0.33) | 1.35(1.31, 1.39) | 0.76(0.53, 0.99) | 2.14(1.70, 2.69) | 0.27(0.21, 0.33) | 1.31(1.23, 1.39) |
| 1920-1924 | 0.75(0.66, 0.84) | 2.12(1.94, 2.31) | 0.27(0.24, 0.29) | 1.31(1.27, 1.34) | 0.71(0.50, 0.91) | 2.02(1.65, 2.49) | 0.23(0.17, 0.28) | 1.25(1.18, 1.33) |
| 1925-1929 | 0.78(0.70, 0.86) | 2.18(2.01, 2.36) | 0.26(0.24, 0.28) | 1.3(1.27, 1.33) | 0.65(0.47, 0.84) | 1.92(1.60, 2.31) | 0.18(0.13, 0.24) | 1.20(1.14, 1.27) |
| 1930-1934 | 0.75(0.67, 0.82) | 2.11(1.96, 2.27) | 0.23(0.21, 0.24) | 1.25(1.23, 1.28) | 0.56(0.40, 0.73) | 1.76(1.49, 2.07) | 0.12(0.08, 0.17) | 1.13(1.08, 1.18) |
| 1935-1939 | 0.66(0.59, 0.73) | 1.93(1.80, 2.07) | 0.16(0.14, 0.17) | 1.17(1.15, 1.19) | 0.44(0.29, 0.58) | 1.55(1.34, 1.79) | 0.03(-0.01, 0.07) | 1.03(0.99, 1.07) |
| 1940-1944 | 0.51(0.44, 0.57) | 1.66(1.56, 1.77) | 0.05(0.04, 0.07) | 1.05(1.04, 1.070) | 0.26(0.14, 0.39) | 1.30(1.15, 1.48) | -0.08(-0.11, -0.05) | 0.92(0.89, 0.96) |
| 1945-1949 | 0.39(0.33, 0.45) | 1.48(1.39, 1.57) | -0.03(-0.04, -0.02) | 0.97(0.96, 0.98) | 0.15(0.04, 0.26) | 1.16(1.04, 1.30) | -0.16(-0.19, -0.13) | 0.85(0.83, 0.88) |
| 1950-1954 | 0.25(0.19, 0.31) | 1.28(1.20, 1.36) | -0.12(-0.13, -0.11) | 0.88(0.88, 0.89) | 0(-0.09, 0.09) | 1.00(0.91, 1.09) | -0.24(-0.26, -0.22) | 0.79(0.77, 0.80) |
| 1955-1959 | 0(-0.07, 0.06) | 1(0.93, 1.06) | -0.25(-0.25, -0.24) | 0.78(0.78, 0.79) | -0.18(-0.26, -0.11) | 0.83(0.77, 0.90) | -0.32(-0.34, -0.3) | 0.73(0.71, 0.74) |
| 1960-1964 | -0.27(-0.33, -0.20) | 0.77(0.72, 0.82) | -0.34(-0.35, -0.34) | 0.71(0.71, 0.71) | -0.34(-0.41, -0.28) | 0.71(0.66, 0.76) | -0.37(-0.38, -0.35) | 0.69(0.69, 0.70) |
| 1965-1969 | -0.49(-0.56, -0.42) | 0.61(0.57, 0.66) | -0.41(-0.41, -0.40) | 0.67(0.66, 0.67) | -0.48(-0.54, -0.42) | 0.62(0.58, 0.66) | -0.39(-0.40, -0.39) | 0.68(0.67, 0.68) |
| 1970-1974 | -0.76(-0.84, -0.68) | 0.47(0.43, 0.51) | -0.45(-0.45, -0.44) | 0.64(0.64, 0.64) | -0.71(-0.78, -0.65) | 0.49(0.46, 0.52) | -0.40(-0.41, -0.4) | 0.67(0.67, 0.67) |
| 1975-1979 | -0.90(-1.01, -0.79) | 0.41(0.36, 0.45) | -0.43(-0.43, -0.42) | 0.65(0.65, 0.66) | -0.87(-0.95, -0.78) | 0.42(0.39, 0.46) | -0.37(-0.37, -0.36) | 0.69(0.69, 0.70) |
| 1980-1984 | -1.14(-1.29, -0.99) | 0.32(0.28, 0.37) | -0.38(-0.38, -0.37) | 0.69(0.68, 0.69) | -0.98(-1.09, -0.87) | 0.38(0.34, 0.42) | -0.27(-0.28, -0.25) | 0.77(0.76, 0.78) |
| 1985-1989 | -1.37(-1.56, -1.18) | 0.25(0.21, 0.31) | -0.30(-0.31, -0.29) | 0.74(0.73, 0.75) | -1.09(-1.23, -0.95) | 0.34(0.29, 0.39) | -0.14(-0.15, -0.12) | 0.87(0.86, 0.89) |
| 1990-1994 | -1.57(-1.83, -1.30) | 0.21(0.16, 0.27) | -0.24(-0.25, -0.22) | 0.79(0.78, 0.80) | -1.22(-1.45, -1.00) | 0.29(0.24, 0.37) | -0.01(-0.03, 0.02) | 0.99(0.97, 1.02) |
| 1995-1999 | -1.75(-2.28, -1.22) | 0.17(0.10, 0.29) | -0.23(-0.26, -0.21) | 0.79(0.77, 0.81) | -1.41(-1.88, -0.94) | 0.24(0.15, 0.39) | 0.07(0.04, 0.10) | 1.07(1.04, 1.11) |
